# Activation of TRIM37 by ATF6 and degradation of ACSL4: inhibiting ferroptosis and propelling cervical cancer progression

**DOI:** 10.1186/s41065-025-00404-9

**Published:** 2025-03-29

**Authors:** Yang Wang, Li Xie, Shiying Jin, YouXiang Hou, Yina Wang

**Affiliations:** 1 Second Department of Thoracic Surgery, Affiliated Tumor Hospital of Xinjiang Medical University, Xinjiang Uyghur Autonomous Region, Urumqi City, 830011 China; 2 First Department of Gynecological Tumor Radiotherapy, Affiliated Tumor Hospital of Xinjiang Medical University, Xinjiang Uyghur Autonomous Region, No. 789 Suzhou East Street, Xinshi District, Urumqi City, 830011 China

**Keywords:** Cervical cancer, Ubiquitination, TRIM37/ATF6/ACSL4, Transcriptional regulation, Ferroptosis, Oncogenesis

## Abstract

**Background:**

Cervical cancer (CC), a prevalent gynecological malignancy, shows high global incidence and mortality. Tripartite motif-containing 37 (TRIM37), a significant ubiquitinating enzyme, is overexpressed in CC, fueling its progression, but its role in ferroptosis here is unknown.

**Methods:**

TRIM37 expression in CC tissues was first predicted using bioinformatics software. Then, RT-qPCR and Western blot were utilized to confirm TRIM37 expression in CC tissues and cells. Subsequently, cellular behaviors were examined by EdU, flow cytometry, and Transwell assay. Besides, ferroptosis-related indicators were detected by using corresponding kits. The dual luciferase reporter assay was conducted to identify the binding between TRIM37 and Activating Transcription Factor 6 (ATF6). Additionally, the Co-IP assay was applied to validate the interaction between TRIM37 and Acyl-CoA Synthetase Long-Chain Family Member 4 (ACSL4). Finally, the functions of TRIM37 in vivo were investigated by establishing a xenograft tumor model.

**Results:**

TRIM37 expression was increased in CC tissues and cells. Silencing TRIM37 suppressed cell malignant behaviors and promoted ferroptosis. ATF6 activated TRIM37 transcription, with TRIM37 upregulation counteracting ATF6 knockdown effects. TRIM37 degraded ACSL4, and silencing ACSL4 reversed TRIM37 knockdown effects. TRIM37 overexpression counteracted ATF6 knockdown’s impact on tumor growth in vivo.

**Conclusion:**

ATF6 regulated the expression of TRIM37, which in turn promoted the ubiquitination and degradation of ACSL4, facilitating the progression of CC.

**Graphical Abstract:**

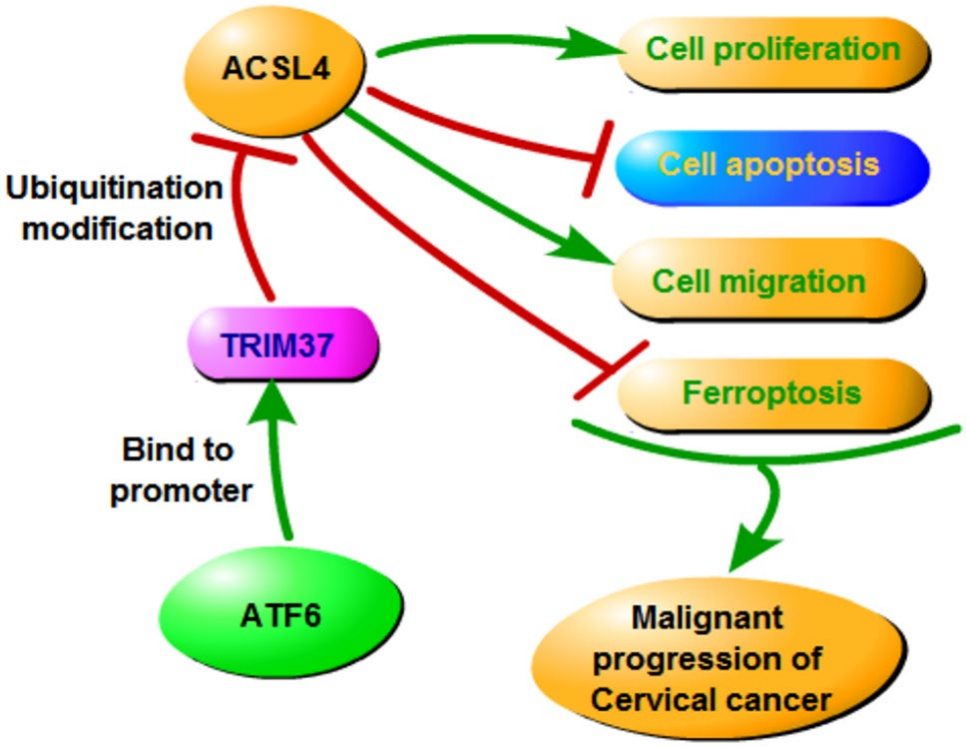

## Highlights

Elevated TRIM37 expression in cervical cancer tissues and cells.

TRIM37 knockdown inhibits proliferation and metastasis, and promots ferroptosis of cervical cancer cells.

ATF6 activation drives TRIM37 transcription, enhancing malignancy in cervical cancer cells.

ACSL4 silencing reverses the effects of TRIM37 knockdown in cervical cancer cells.

TRIM37 overexpression counteracts ATF6 knockdown’s impact on tumor growth in vivo.

## Introduction

Cervical cancer (CC) ranks as the fourth most prevalent cancer among women globally, following breast, colorectal, and lung cancers [50]. According to GLOBOCAN 2020, approximately 604,000 new CC cases are diagnosed globally each year, with 342,000 associated deaths [36]. The primary cause is the uncontrolled proliferation of abnormal CC cells [20]. These aberrant cells, unregulated by the body’s normal mechanisms, can invade the adjacent cervical tissues and metastasize to other body parts via the lymphatic system or bloodstream [14, 39]. Conventional CC treatments have significant drawbacks. Surgical treatments, constrained by the disease stage, often fail to achieve a cure in in mid to advanced cases and are accompanied by multiple postoperative complications. Radiotherapy targets tumors but invariably affects surrounding healthy tissues. Chemotherapy induces systemic side effects and often leads to drug resistance [4, 12, 46]. Given these challenges, it is imperative to investigate new therapeutic targets, overcome existing limitations, and enhance the overall treatment paradigm.

Tripartite motif containing 37 (TRIM37), a key ubiquitination enzyme, is the focus of our research. As a member of the TRIM family with a ring finger structural domain, it functions as an E3 ubiquitin ligase and is crucial in the development of cancer [3, 11]. A previous study confirmed that TRIM37 showed elevated expression levels in breast cancer subtypes. Research findings have shown that TRIM37 is closely related to chemotherapy resistance and metastasis in triple-negative breast cancer (TNBC) [40]. Additionally, TRIM37 enhances the invasion and metastasis of cancer cells, such as those in colorectal, hepatocellular, non-small-cell lung cancer, pancreatic and gastric cancers [8, 16, 18, 19, 49]. However, TRIM37’s functions in the development of CC and the related molecular mechanisms are yet to be elucidated.

Ferroptosis, a newly discovered type of non-apoptotic regulated cell death, is typified by the accumulation of iron-dependent lipid peroxides, which ultimately results in lethal cell damage. Furthermore, recent research has indicated that the induction of ferroptosis by some drugs can effectively impede tumor progression [6, 10, 33, 41]. The progression of CC is closely associated with iron regulation and oxidative stress [5]. Due to the important role of iron in the metabolism of CC cells, it is worthwhile to explore the role of ferroptosis in the pathogenesis of CC [5]. Existing clues suggest that the abnormal expression of some ubiquitination enzymes, like TRIM37, may interfere with iron metabolism and lipid peroxidation levels in cancer cells, implying its involvement in the regulation of ferroptosis in cervical cells [28, 32, 51].

Activating transcription factor 6 (ATF6) is a protein located in the endoplasmic reticulum (ER) and belongs to the leucine zipper family. It is known for transducing signals associated with ER stress [1, 15]. In CC, the activation of ATF6 regulates cell growth and migration while inhibiting apoptosis and autophagy through the MAPK pathway [23]. From a molecular perspective, it remains elusive whether a regulatory association exists between ATF6 and TRIM37 in CC and whether the ubiquitination modifications mediated thereby are implicated in the process of ferroptosis in cervical cells. ACSL4 is a lipid-metabolizing enzyme that can promote ferroptosis. It is an important pharmacological target for treating ferroptosis-related diseases [17]. ACSL4 promotes the build-up of lipid intermediates during ferroptosis. It has been identified not only as a biomarker for ferroptosis but also as a factor contributing to this process [48]. Jiang et al.‘s research indicates that oleanolic acid induces ferroptosis in Hela cells, During which, the expression of ACSL4 is upregulated and significant ferroptosis-characteristic changes occur, such as an increase in intracellular iron ion concentration, accumulation of lipid peroxides, and a decrease in GPX4 activity. This demonstrates that ACSL4 is involved in the ferroptosis process induced by oleanolic acid [44]. ACSL4-mediated ferroptosis is a molecular event that exerts a crucial regulatory function in cancer development. Research has shown that the ubiquitination and degradation of ACSL4 ultimately induce tumor cells’ resistance to ferroptosis. As described in the research on colorectal cancer by Chen et al., CYP1B1 inhibits ferroptosis by degrading ACSL4 and promotes the progression of colorectal cancer [7]. Nevertheless, it remains unknown how the ubiquitination modification mediated by TRIM37 affects the key molecules of ferroptosis and whether it promotes or inhibits it. These uncertainties highlight the necessity for further research on the association between the two and related mechanisms.

Herein, the focus of this study is to uncover the regulatory mechanism of TRIM37-mediated ubiquitination modification in CC and to elucidate the role that ferroptosis resulting from ubiquitination modification plays in the development of CC, providing a theoretical basis and potential research directions for subsequent studies on the molecular mechanism exploration and the treatment of CC.

## Materials and methods

### Cell lines

Ect1/E6E7 cells were cultured in the Keratinocyte Serum-Free Medium (K-SFM) (Thermo Fisher Scientific, Rockville, MD, USA) containing 0.05 mg/mL Bovine Pituitary Extract (BPE) (Corning, Tewksbury, MA, USA) and 5 ng/mL Recombinant Epidermal Growth Factor (EGF) (Beyotime, Shanghai, China). SiHA and HeLa cells, maintained in Minimum Essential Medium (MEM) (Thermo Fisher Scientific) supplemented with 10% Fetal Bovine Serum (FBS) (Thermo Fisher Scientific), were all procured from the China Center for Type Culture Collection (Wuhan, China). Me180 cells were sourced from Chuan Qiu Biotechnology (Shanghai, China) and cultured in McCoy’s 5 A medium (Pricella) supplemented with 10% FBS (Thermo Fisher Scientific). C-33 A cells, purchased from Pricella (Wuhan, China), were grown in MEM (NEAA) (Pricella) containing 10% FBS (Thermo Fisher Scientific) and 1% penicillin/streptomycin (Invitrogen, Carlsbad, CA, USA).

### Clinical samples

Thirty-four pairs of CC specimens and matched adjacent noncancerous tissues were collected from patients who underwent resection surgery at Affiliated Tumor Hospital of Xinjiang Medical University. Before collecting samples, no patients had undergone preoperative chemotherapy or radiotherapy. The research protocol was approved by the Ethics Committee of the Affiliated Tumor Hospital of Xinjiang Medical University, and all patients provided their written consent[Approval Number: K-2024024].

### Real-time quantitative reverse transcription polymerase chain reaction (RT-qPCR)

Total RNAs were isolated using TRIzol reagent (Invitrogen). The cDNA obtained by using the Transcriptor First Strand cDNA Synthesis Kit (Roche, Vilvoord, Brussels, Belgium) was used as a template. Specific primers were synthesized by the Beijing Genomics Institute (BGI, Shenzhen, China). Amplification reactions were performed with SYBR Green PCR Master Mix (Ambion, Carlsbad, CA, USA) using a Real-Time PCR Detection System (Bio-Rad, Shanghai, China). The primer sequences were as follows: TRIM37 (5’-3’): Forward: ACCAGTTGCGGTCTTGTAGT; Reverse: GTCTGGTGGAACAGGAGTGG. ACSL4 (5’-3’): Forward: AGCTTTCTCAGTTGAAGGCGTA; Reverse: TAACAAGTGGACAGGCAGCA. GAPDH (5’-3’): Forward: GGAGCGAGATCCCTCCAAAAT; Reverse: GGCTGTTGTCATACTTCTCATGG.

### Western blot

Proteins were extracted from the CC tissues and cells using a protein extraction kit (Beyotime, Shanghai, China). Then, 10 µg of proteins were mixed with the loading buffer to prepare the sample. They were separated by Sodium dodecyl sulfate-polyacrylamide gel electrophoresis (SDS-PAGE). Next, protein bands were transferred onto a PVDF membrane (GE Healthcare, Piscataway, NJ, USA). 5% skimmed milk was used to block the non-protein-binding sites on the membranes. Subsequently, the membranes were incubated overnight at 4℃ with primary antibodies (TRIM37 1:500, Cat: ab264190Abcam, Cambridge, UK; ATF6 1:500, Cat: ab37149, Abcam; ACSL4 1:500, Cat: ab205199, Abcam; GAPDH 1:500, Cat: ab9485, Abcam), followed by incubation with secondary antibodies (Rabbit Anti-Mouse IgG H&L (HRP) 1:2000, Cat: ab6728, Abcam; Goat Anti-Rabbit IgG H&L (HRP) 1:2000, Cat: ab6721, Abcam) at room temperature for 1 h. Chemiluminescence intensity was analyzed using RapidStep ECL Reagent (Millipore Corp., Billerica, MA, United States). For protein quantification using Image J software, Western blot image was first imported and pre-processed. The integrated density values of the GAPDH and target protein bands were measured in sequence. After background correction, the corrected integrated density value of the target protein was divided by that of GAPDH to obtain the relative expression level of the target protein. The data were recorded and statistically analyzed.

### Cell transfection

SiHa and HeLa cells were inoculated in 6-well plates at a density of 2 × 10^5^ cells/well, 12 h before transfection. The small interference RNAs (siRNAs) targeting TRIM37 (si-TRIM37#1 and si-TRIM37#2) and ATF6 (si-ATF6), along with the short hairpin RNA (shRNA) targeting ATF6 (sh-ATF6), were designed by GenePharma (Shanghai, China) and GeneChem (Shanghai, China). Non-targeted siRNAs or shRNAs were used as the negative control (si-NC and sh-NC). The overexpression vector of TRIM37 was constructed by cloning TRIM37 gene into pcDNA3.1 vector (Invitrogen) (labeled as OE-TRIM37), while the empty pcDNA3.1 plasmids served as the negative control (OE-NC). The siRNAs, shRNAs and overexpression vectors were transfected into the SiHa and HeLa cells using Lipofectamine 2000 (Invitrogen) following the manufacturer’s instructions.

### 5‐Ethynyl-2’deoxyuridine (EdU) assay

The BeyoClick™ EdU Cell Proliferation Kit with Alexa Fluor 488 (Beyotime) was utilized in this assay according to the methods described in previous literature [25]. Cells were seeded into a 6-well plate. The EdU working solution (20 µM), pre-warmed to 37℃, was then added to the culture plate, ensuring the added volume was equal to the medium in the wells. After 2 h, the cells were fixed using 4% paraformaldehyde (Beyotime) at room temperature for 15 min. Then, they were permeabilized using 0.3% Triton X-100 (Biosharp, Beijing, China). The Click reaction solution was added, and the nuclei were stained with DAPI (Thermo Fisher Scientific) at room temperature for 5 min. Finally, EdU-positive cells were analyzed using an inverted fluorescence microscope (Olympus, Tokyo, Japan).

### Flow cytometry

The ANNEXIN V-FITC/PI Apoptosis Detection Kit (Beyotime) was employed to determine cell apoptosis. As described in the instructions, SiHa and HeLa cells were first collected into centrifuge tubes by centrifugation. The cells were resuspended in 1× binding buffer to reach a concentration of 5 × 10^6^/mL. Subsequently, 100 µL of cell suspension was pipetted into a 5 mL flow tube (with 5 µL of AnnexinV/FITC) and incubated at room temperature away from light. After 5 min of incubation in the dark, 5 µL of propidium iodide (PI) and 400 µL of PBS were added and the apoptotic cells were immediately analyzed by flow cytometry.

### Transwell assay

Transwell insert chambers with a filter pore size of 8 μm for 24-well plate (Corning Inc., Corning, NY, USA) were used to detect cell migration [30]. SiHa and HeLa cells (1 × 10^5^) were seeded into the upper chamber with 200 µL of FBS-free medium. Meanwhile, 600 µL of complete medium (with 10% FBS) was added to the bottom chambers. After 48 h of incubation at 37℃, the cells that migrated to the bottom chambers were fixed using 4% paraformaldehyde (Beyotime) at room temperature. After 20 min of fixation, the cells were stained with 0.1% crystal violet (Beyotime). The migrated cells were then observed and photographed using a microscope.

### Evaluation of ferroptosis

Ferroptosis-related indicators were measured to analyze the ferroptosis of CC cells. The Fe^2+^ contents were measured using the Ferrous ion Content Detection Kit (Solaribio, Beijing, China) [47]. After sonication and centrifugation, the supernatant was placed on ice. Samples, standards and reagents were added in accordance with the instructions. The absorbance at 593 nm was measured using a Microplate Reader and the Fe^2+^ contents were calculated according to the standard curve. Following the description in previous literature [13], reactive oxygen species (ROS) levels were measured using the Reactive Oxygen Species Assay Kit (Beyotime). SiHa and HeLa cells were collected and then loaded with the probe 2,7-dichlorofluorescin diacetate (DCFH-DA). The final results were analyzed using a fluorescence microscope. For GSH measurement, the Reduced Glutathione (GSH) Colorimetric Assay Kit (Elabscience, Wuhan, China) was used. The procedures were carried out following the descriptions of previous researchers [43]. Finally, the optical density (OD) value at 405 nm was detected, and GSH levels were computed following the standard curve, OD value, and the protein concentrations of samples.

### Bioinformatic analysis

To explore the transcription factors that TRIM37 might bind to, the Genecards database (https://www.genecards.org/.) was utilized. Subsequently, the Jasper website (https://jaspar.genereg.net/) was applied to explore the binding sites between ATF6 and the TRIM37 promoter. After selecting the appropriate species database, ‘ATF6’ was entered to search for its information. Then, the TRIM37 promoter sequence was retrieved from the GenBank database (https://www.ncbi.nlm.nih.gov/genbank/) and then imported into the Jasper analysis module. After the prediction analysis was initiated, the results regarding the possible binding sites of ATF6 on the TRIM37 promoter were generated.

### Dual-luciferase reporter assay

The pGL3-basic-TRIM37-WT/MUT was constructed by cloning wild-type (WT) or mutant-type (MUT) TRIM37 sequences into the pGL3-basic vector (Promega, Madison, WI, USA). The SiHa and HeLa cells transiently transfected with si-NC or si-ATF6 were treated with the pGL3-basic-TRIM37-WT/MUT vector and the pRL-TK vector encoding Renilla luciferase (serving as the internal control), using the jetPRIMETM-DNA-siRNA transfection reagent (Polyplus, Strasbourg, France). After a 48-hour incubation period, the cells were lysed and the dual-luciferase reporter assay was performed to estimate the luciferase activity, aiming to determine the transcriptional regulatory relationship between relevant genes, using the Dual-Luciferase^®^ Reporter Assay system (Promega).

### Co-imunoprecipitation (Co-IP) assay

Co-IP assay was conducted to determine the interaction between ACSL4 and TRIM37. SiHa and HeLa cells were lysed with cell lysis buffer (containing 1% Triton X-100, 150 mM NaCl, and 50 mM Tris-HCl, pH 7.4, with protease inhibitors and phosphatase inhibitors), and one portion of the cell lysate was reversed as an input sample, while the other was separately incubated with anti-TRIM37 (Cat: ab264190, Abcam, Cambridge, UK) and anti-ACSL4 (Cat: ab205199, Abcam) respectively overnight at 4℃. The next day, protein A/G agarose beads pre-equilibrated with lysis buffer were added to each antibody-lysate incubation system, and incubation was continued at 4℃ for 2 h. After washing, proteins were eluted and protein interactions were verified by Western blot.

### Analysis of protein stability

si-NC and si-TRIM37#1 were transfected into SiHa and HeLa cells. The transfected cells were treated with 5 µg/mL cycloheximide (CHX) (Yeasen, Shanghai, China), a protein synthesis inhibitor, at different time-points (0, 5, 10, 20, and 25 h). Total proteins in the cells were extracted at these time points. Using GAPDH as an internal control, ACSL4 expression levels were tested by Western blot, and the changes of ACSL4 protein over time in different treatment groups were analyzed.

### Detection of ubiquitinated protein

Cells transfected with si-NC and si-TRIM37#1 were harvested and lysed. Cell lysates were immunoprecipitated with anti-ACSL4 (Cat: ab205199, Abcam) to enrich protein complexes interacting with ACSL4 protein. At the same time, anti-IgG (Cat: ab133470, Abcam) (as a control for non-specific binding) and input sample (total cell lysate not immunoprecipitated) were set as controls. The protein complexes obtained by IP and control samples were analyzed by immunoblotting (IB) to determine the presence and relative amounts of target proteins. The ubiquitinated protein levels were detected using anti-ubiquitin (Ub) (Cat: ab134953, Abcam), ACSL4 protein levels were detected using ACSL4 antibody (Cat: ab205199, Abcam), and anti-GAPDH (Cat: ab9485, Abcam) was used as an internal reference to test the consistency of sample loading.

### Establishment of tumor xenograft model

To explore the role of TRIM37/ATF6 in the in vivo tumor growth of CC, tumor xenograft models were constructed. Female BALB/c (4–5 weeks) nude mice were purchased from Aniphe Biolaboratory Inc. (Jiangsu, China), housed in suitable cages and given free access to food and water. The mice were randomly divided into three groups (*n* = 5 per group): sh-NC, sh-ATF6 and sh-ATF6 + OE-TRIM37. Recombinant lentivirus was used to engineer SiHa cells stably expressing sh-ATF6 and sh-ATF6 + OE-TRIM37, and corresponding control cells (sh-NC). Each group of mice received a subcutaneous inoculation of these cells at a density of 1 × 10^6^ cells on the right flank. Tumor sizes were measured every four days, and the tumor volume was calculated using the formula: volume (mm^3^) = width^2^ × length/2. On the 28th day, the cervical dislocation method was used to sacrifice the mice. The tumors were excised, weighed, and photographed. The aforementioned animal experiments were performed in accordance with the Guidelines for the Care and Use of Laboratory Animals released by Affiliated Tumor Hospital of Xinjiang Medical University and approved by the Ethics Committee of Affiliated Tumor Hospital of Xinjiang Medical University[Approval Number: K-2024024].

### Immunohistochemistry (IHC)

To confirm the expression of TRIM37 and ACSL4 in tumors, IHC was performed. The isolated tumor tissues were fixed in 10% neutral formalin for 12 h. Following dehydration and embedding, the tissues were sectioned into 4-µm slices. Subsequently, the steps of dewaxing, hydration and antigen retrieval were carried out. Blocking was performed using 5% BSA (Solarbio). One hour later, the slices were successively incubated with the primary antibody and the secondary antibody. Color development was carried out using the DAB Horseradish Peroxidase Color Development Kit (Beyotime). After counterstaining, dehydration and mounting, the staining situation was observed and recorded under the microscope.

### Statistical analysis

All experiments were performed at least three times, with results expressed as the mean ± standard deviation (SD). The Graphpad Prism 8.0.1 software was applied for data processing and analysis. The normality of the data was evaluated using the Shapiro-Wilk normality test. For analyses of two groups, the unpaired *t*-test was used to compare independent samples. While for the analyses involving more than two groups, one-way and two-way ANOVA was employed for analysis. A *P*-value of < 0.05 was considered statistically significant.

## Results

### TRIM37 exhibited high expression in CC cells and tissues

**Fig. 1:**
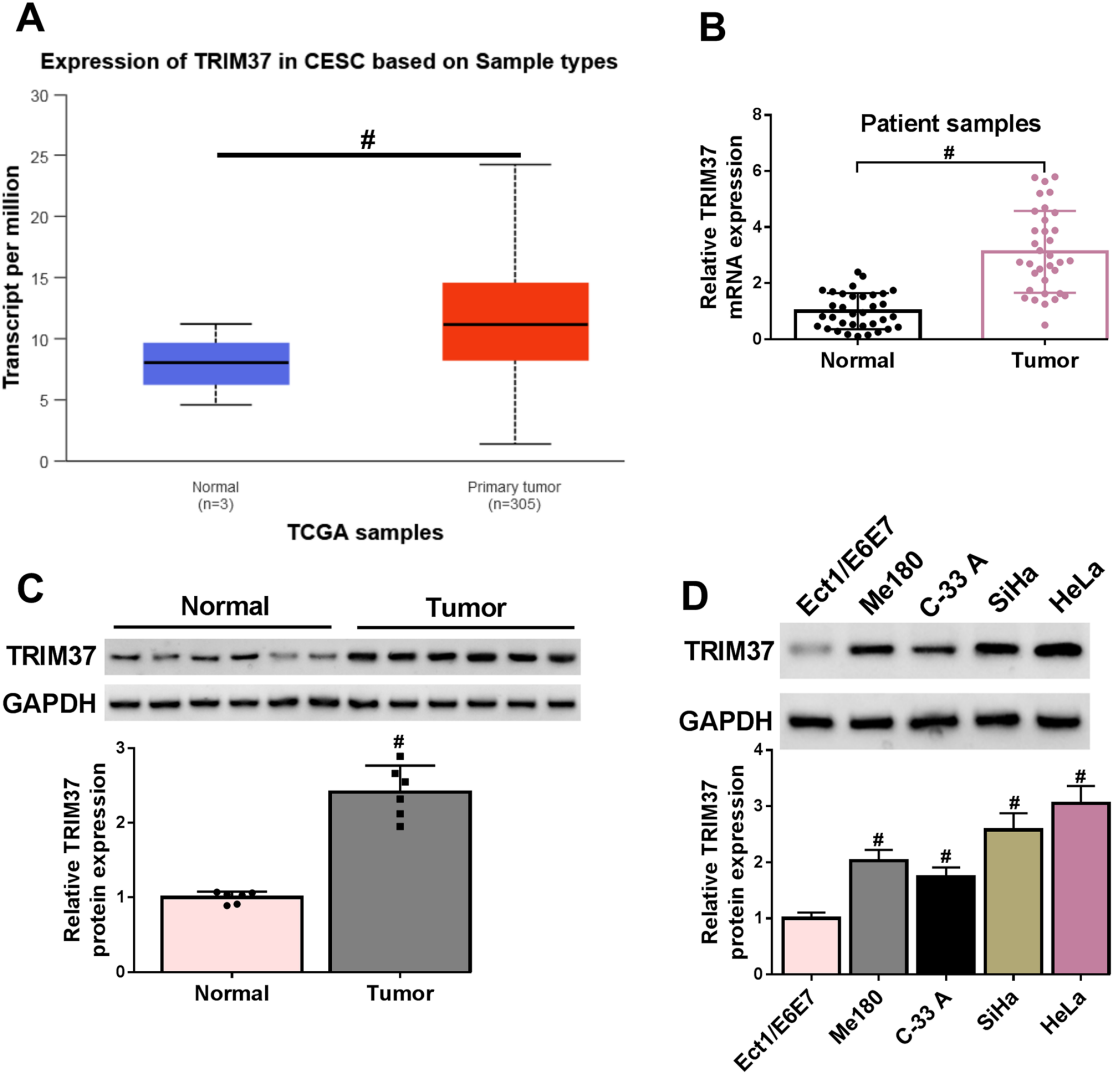
The expression of TRIM37 was upregulated in CC. (**A**) UALCAN database showed TRIM37 expression in CESC based on TCGA samples (Normal: *n* = 3, Primary tumor: *n* = 305). (**B**) The expression levels of TRIM37 mRNA in 34 pairs of normal and tumor tissues were detected by RT-qPCR. (**C**) Western blot analysis of TRIM37 protein levels in 6 normal tissues and 6 tumor tissues. (**D**) The expression levels of TRIM37 protein in normal human cervical cells (Ect1/E6E7) and human CC cells (Me180, C-33 A, SiHa, and HeLa). # *P* < 0.05

The TCGA database was used to show that the expression of the TRIM37 gene in cervical squamous cell carcinoma (CESC) was notably higher in primary tumor samples (*n* = 305) compared to normal samples (*n* = 3) (Fig. 1A). Then, RT-qPCR assay was used to determine TRIM37 expression levels in 34 pairs of CC tissues, which showed a higher level in the tumor tissue than in normal tissues (Fig. 1B). Meanwhile, TRIM37 protein expression levels in tissues and cells (Ect1/E6E7, Me180, C-33 A, SiHa, and HeLa) of CC were examined by Western blot. TRIM37 protein was expressed at higher levels in both CC tissues and cells than in normal tissues and normal cervical cells (Fig. 1C-D). Collectively, TRIM37 was upregulated both in CC tissues and cells.

### Silencing TRIM37 suppressed malignant invasion and promoted ferroptosis of CC cells

**Fig. 2:**
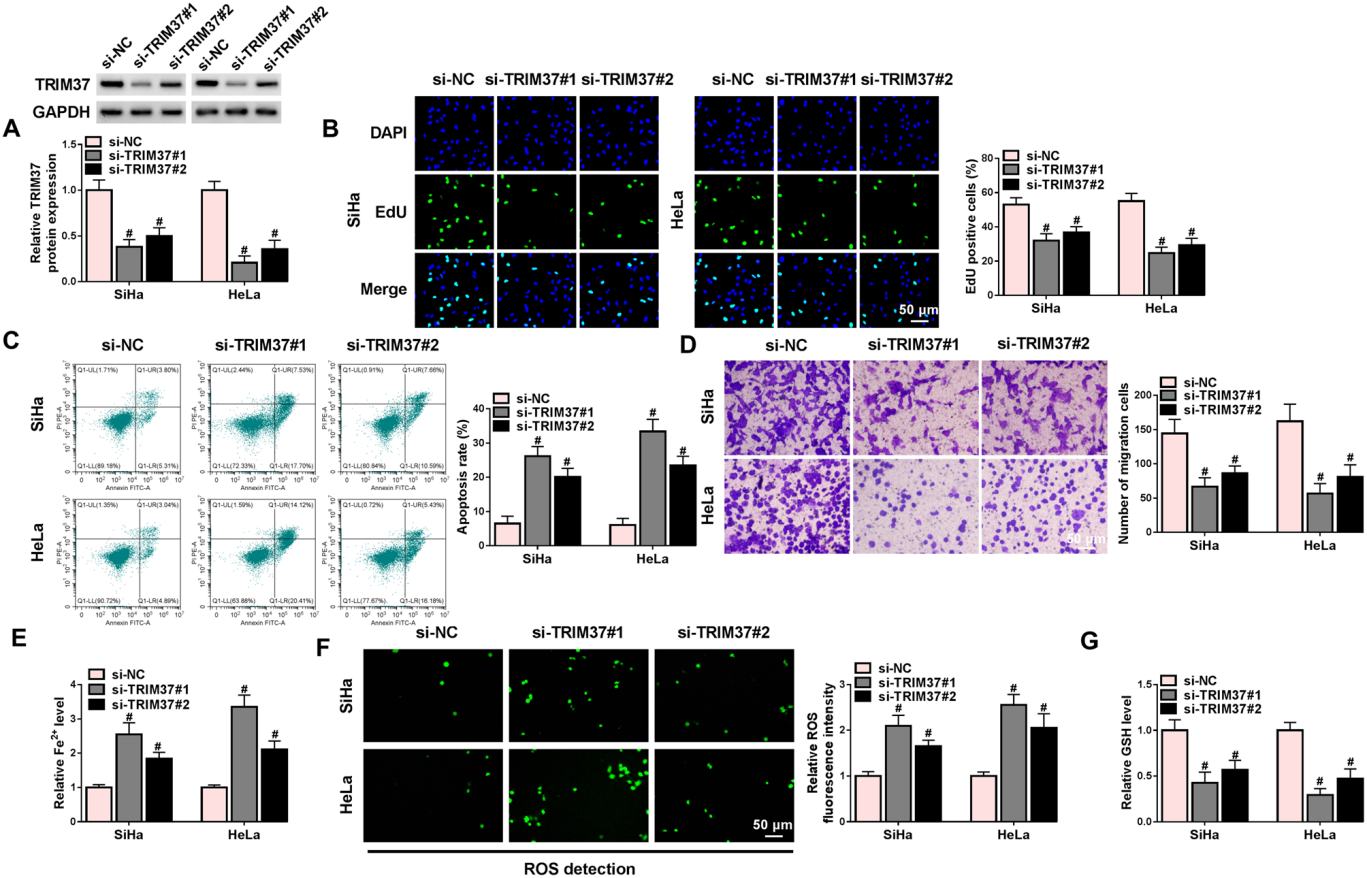
Silencing TRIM37 inhibited the proliferation and metastasis of CC cells and promotes ferroptosis. SiHa and HeLa cells were transfected with si-NC, si-TRIM37#1, and si-TRIM37#2. (**A**) Western blot analysis was utilized to confirm the transfection efficiency. (**B**) The proliferation of CC cells in each group was determined by EdU assay. (**C**) The flow cytometry was employed to evaluate the apoptosis of CC cells. (**D**) The migration capability was evaluated by using Transwell assay. (**E**-**G**) The levels of Fe^2+^, ROS and GSH were measured by using corresponding kits. # *P* < 0.05

To clarify whether TRIM37 impacted the biological characteristics of CC, a series of loss-of-function assays were carried out. First, the interference efficiency was determined by Western blot. TRIM37 expression levels in both SiHa and HeLa cells were decreased after transfection with si-TRIM37, among which si-TRIM37#1 exhibited the highest knockdown efficiency (Fig. 2A). The EdU assay indicated that TRIM37 downregulation markedly inhibited the proliferation of CC cells (Fig. 2B). Additionally, the flow cytometry and Transwell assay were conducted to estimate apoptosis and migration of CC cells. The results suggested that the TRIM37 downregulation promoted apoptosis and constrained migration (Fig. 2C-D). Next, the indicators related to ferroptosis were measured. Elevated levels of Fe^2+^ and ROS, and decreased GSH levels due to transfection of si-TRIM37 were observed (Fig. 2E-G). In summary, the knockdown of TRIM37 impeded proliferation and migration, whereas it triggered cell apoptosis and ferroptosis of CC cells.

### Transcription factor ATF6 activated transcriptional regulation of TRIM37

**Fig. 3:**
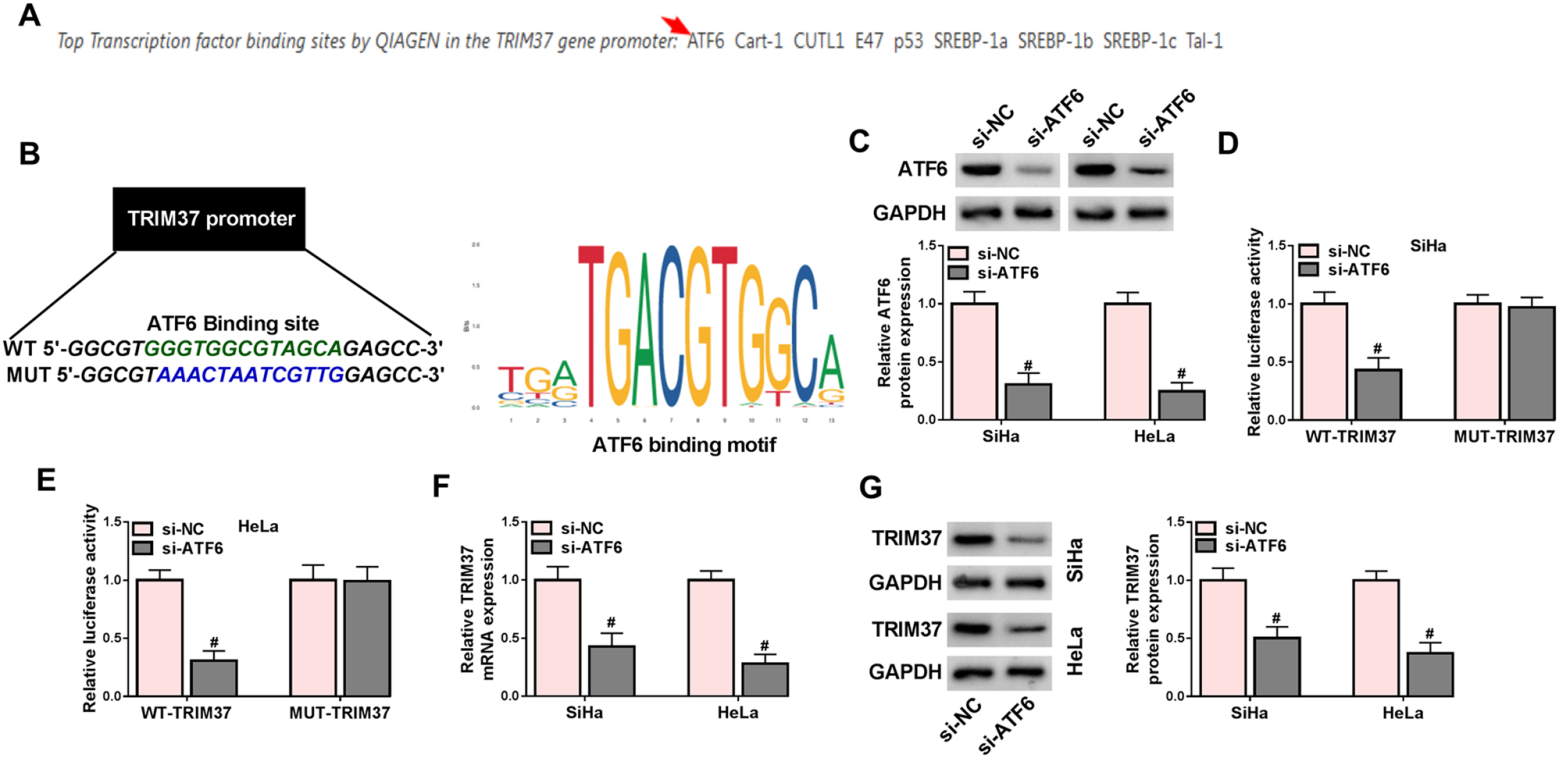
The transcription factor ATF6 could activate the transcriptional regulation of TRIM37. (**A**) The Genecards database was used to predict the transcription factors that TRIM37 is most likely to bind to. (**B**) The binding sites between ATF6 and the TRIM37 promoter were predicted by the Jaspar website. (**C**) The expression levels of ATF6 in SiHa and HeLa cells transfected with si-NC and si-ATF6 were detected by Western blot. (**D**-**E**) The dual-luciferase reporter assay was used to verify the binding of ATF6 to the TRIM37 promoter. (**F**) RT-qPCR assay was applied to analyze the TRIM37 mRNA expression levels in SiHa and HeLa cells transfected with si-NC and si-ATF6. (**G**) The TRIM37 protein levels in SiHa and HeLa cells treated with si-NC and si-ATF6 were detected by Western blot. # *P* < 0.05

The Genecards database was used to predict the transcription factors likely to bind to TRIM37, identifying ATF6 as a candidate (Fig. 3A). In addition, Jasper website showed that there were binding sites between ATF6 and TRIM37 promoter (Fig. 3B). Then, ATF6 was knocked down. Western blot results illustrated that ATF6 protein levels in SiHa and HeLa cells transfected with si-ATF6 were decreased in contrast to those in the control group (Fig. 3C). Then, the TRIM37 promoter was fused with the reporter gene to determine the binding of ATF6. The dual-luciferase activity assay presented that, for WT-TRIM37, si-ATF6-transfected SiHa and HeLa cells had considerably lower activity compared with si-NC-transfected cells. For MUT-TRIM37, both si-NC and si-ATF6 groups had similar activity levels with no significant difference (Fig. 3D-E). Moreover, with the knockdown of ATF6, the expression of TRIM37 at both the mRNA and protein levels was lowered (Fig. 3F-G). Taken together, ATF6 bound to the TRIM37 promoter thereby expediting the expression of TRIM37.

### Overexpression of TRIM37 mitigated the effect of ATF6 knockdown on the aggressiveness of CC cells

**Fig. 4:**
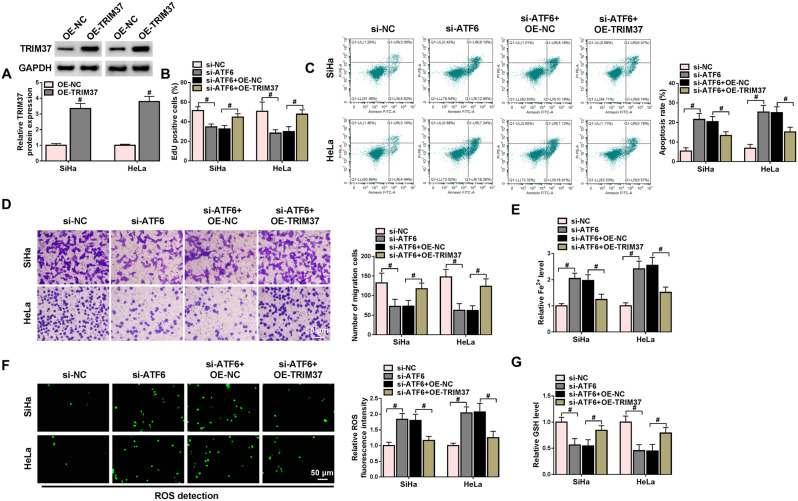
Overexpression of TRIM37 could reverse the effect of ATF6 knockdown on CC cells. SiHa and HeLa cells were transfected with si-NC, si-ATF6, si-ATF6 + OE-NC, and si-ATF6 + OE-TRIM37. (**A**) The overexpression efficiency of TRIM37 was verified by Western blot. (**B**) EdU assay was utilized to analyze the proliferation of CC cells. (**C**-**D**) The apoptosis and migration of CC cells in different groups were monitored by using flow cytometry and Transwell assay. (**E**-**G**) The ferroptosis was estimated by detecting the levels of Fe^2+^, ROS, and GSH in CC cells. # *P* < 0.05

Subsequently, the role played by the interaction between ATF6 and TRIM37 in CC was further elucidated. Western blot analysis was conducted to validate the overexpression of TRIM37 in CC cells. TRIM37 expression was elevated after cells were transfected with OE-TRIM37 (Fig. 4A). As shown by the EdU assay, flow cytometry, and Transwell assay results, the decrease in EdU-positive cells and the number of migrated cells and the increase in apoptotic cells caused by ATF6 knockdown were restored by TRIM37 overexpression (Fig. 4B-D). Besides, the levels of Fe^2+^, ROS and GSH were measured to analyze the ferroptosis of CC cells. Compared with the si-ATF6 group, the Fe^2+^ and ROS levels of CC cells in the si-ATF6 + OE-TRIM37 group were lower, while the level of GSH was higher, indicating that the effect of increased ferroptosis caused by ATF6 knockdown was reversed by the upregulation of TRIM37 (Fig. 4E-G). To sum up, the downregulation of ATF6 constrained the malignant invasion and ferroptosis of CC cells. Nevertheless, these effects were abolished by TRIM37 overexpression.

### The ubiquitinating enzyme TRIM37 degraded ACSL4

**Fig. 5:**
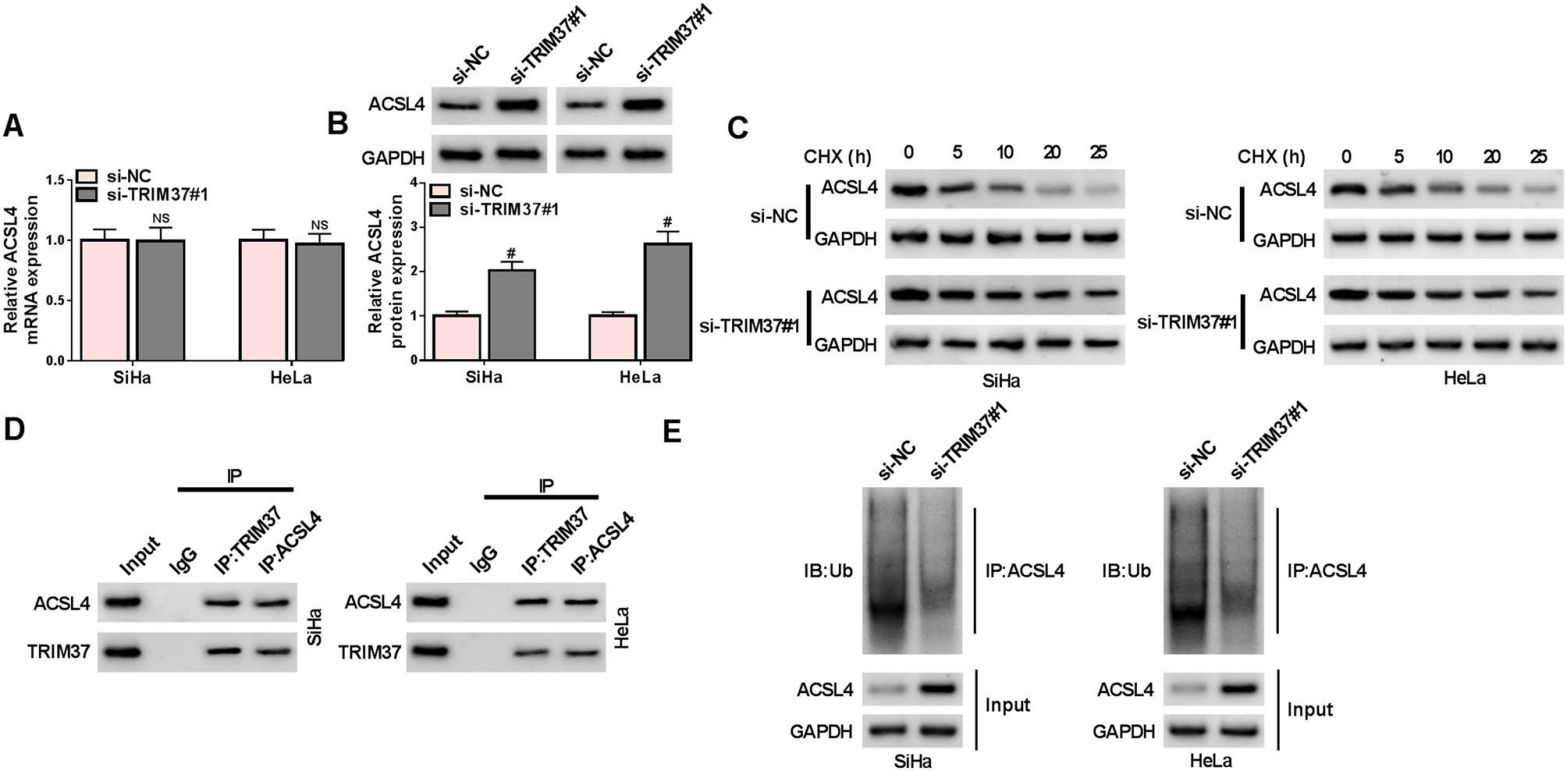
The ubiquitin ligase TRIM37 could degrade ACSL4. The SiHa and HeLa cells were transfected with si-NC and si-TRIM37#1 (**A**-**B**) The expression levels of ACSL4 mRNA and protein in CC cells were examined by RT-qPCR and Western blot. (**C**) The CC cells were treated with CHX, the ACSL4 protein levels were detected by Western blot at specific time points (0, 5, 10, 20, 25 h). (**D**) Co-IP assay was used to demonstrate the interaction between TRIM37 and ACSL4. (**E**) IB and IC were applied to detect the levels of ubiquitinated proteins and ACSL4 protein. # *P* < 0.05

ACSL4 is an important iron death-promoting protein. However, whether ubiquitination was involved in modifying ACSL4 remained unknown. Therefore, the following experiment was conducted to probe whether ACSL4 could be modified by TRIM37 through ubiquitination. RT-qPCR and Western blot confirmed that TRIM37 affected the protein level of ACSL4 without impacting its mRNA level, suggesting that TRIM37 modulated ACSL4 expression at the protein level (Fig. 5A-B). Next, CHX was used to treat SiHa and HeLa cells. The Western blot results in CC cells suggested that as the CHX treatment time increased from 0 to 25 h, the ACSL4 protein level in the si-NC group decreased gradually, while the decrease in the si-TRIM37#1 group was much slower, indicating that TRIM37 silencing could slow down ACSL4 protein degradation (Fig. 5C). Additionally, the Co-IP assay illustrated that TRIM37 could bind to ACSL4 (Fig. 5D). Next, the relationship between ubiquitination modification and the expression level of ACSL4 was assessed. Upon examination, it came to light that following the knockdown of TRIM37, there was a notable decline in the ubiquitination level of ACSL4, and the protein expression level of ACSL4 witnessed an increase. This finding implied that TRIM37 facilitated the degradation of ACSL4 by means of ubiquitination modification (Fig. 5E). In conclusion, TRIM37 diminished the stability of ACSL4 protein through ubiquitination modification.

### Downregulation of ACSL4 counteracted the consequences of TRIM37 knockdown on CC cells

**Fig. 6:**
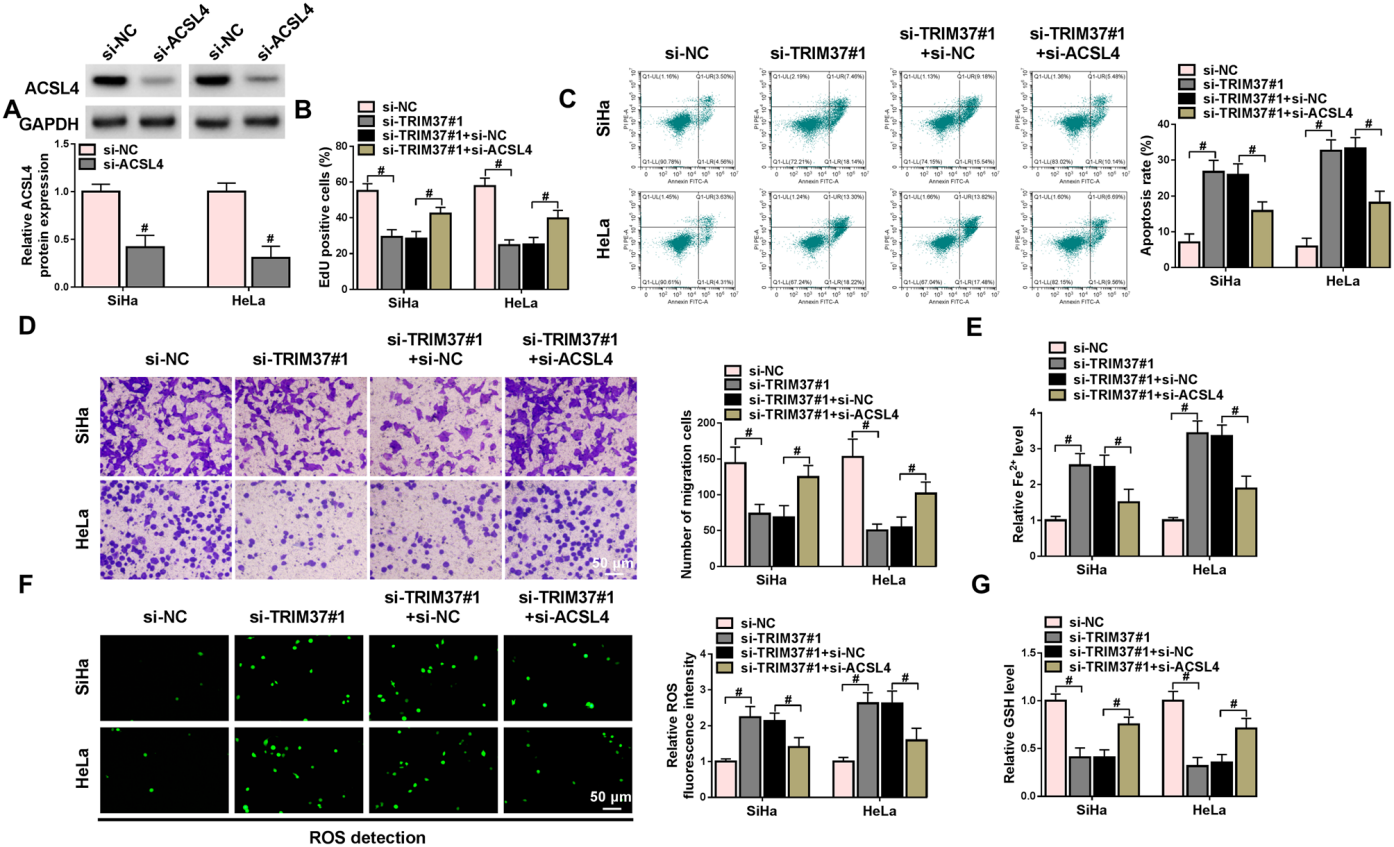
Knockdown of ACSL4 overturned the effects of silencing TRIM37 on CC cells. SiHa and HeLa cell were divided into four groups: the si-NC group, the si-TRIM37# group, the si-TRIM37#1 + si-NC group, and the si-TRIM37#1 + si-ACSL4 group. (**A**) The expression level of ACSL4 in CC cells transfected with si-NC and si-ACSL4 was detected using Western blot. (**C**-**D**) The EdU assay, flow cytometry, and Transwell assay were applied to evaluate the proliferation, apoptosis, and migration of CC cells. (**E**-**G**) Ferroptosis-related indicators (Fe^2+^, ROS, and GSH) were measured using corresponding kits to analyze ferroptosis of CC cells. # *P* < 0.05

Accordingly, whether TRIM37 performed its role in CC via targeting ACSL4 was explored. As shown by Western blot analysis, the ACSL4 expression level in the si-ACSL4 group was lower than that of the control group (Fig. 6A). The suppression of cell proliferation, apoptosis, and migration caused by TRIM37 downregulation was reversed by the downregulation of ACSL4 (Fig. 6B-D). Consistently, the elevation of Fe^2+^ and ROS levels and the decline of GSH level caused by TRIM37 knockdown were likewise reversed by ACSL4 knockdown, suggesting the regulatory role of ACSL4 in cellular homeostasis affected by TRIM37 (Fig. 6E-G). Based on the above, the suppressive influence of TRIM37 on the malignant proliferative trait of CC cells and its promoting effect on ferroptosis could be reverted by the downregulation of ACSL4.

### Upregulation of TRIM37 undermined the inhibitory effect of ATF6 knockdown on tumor growthin vivo

**Fig. 7:**
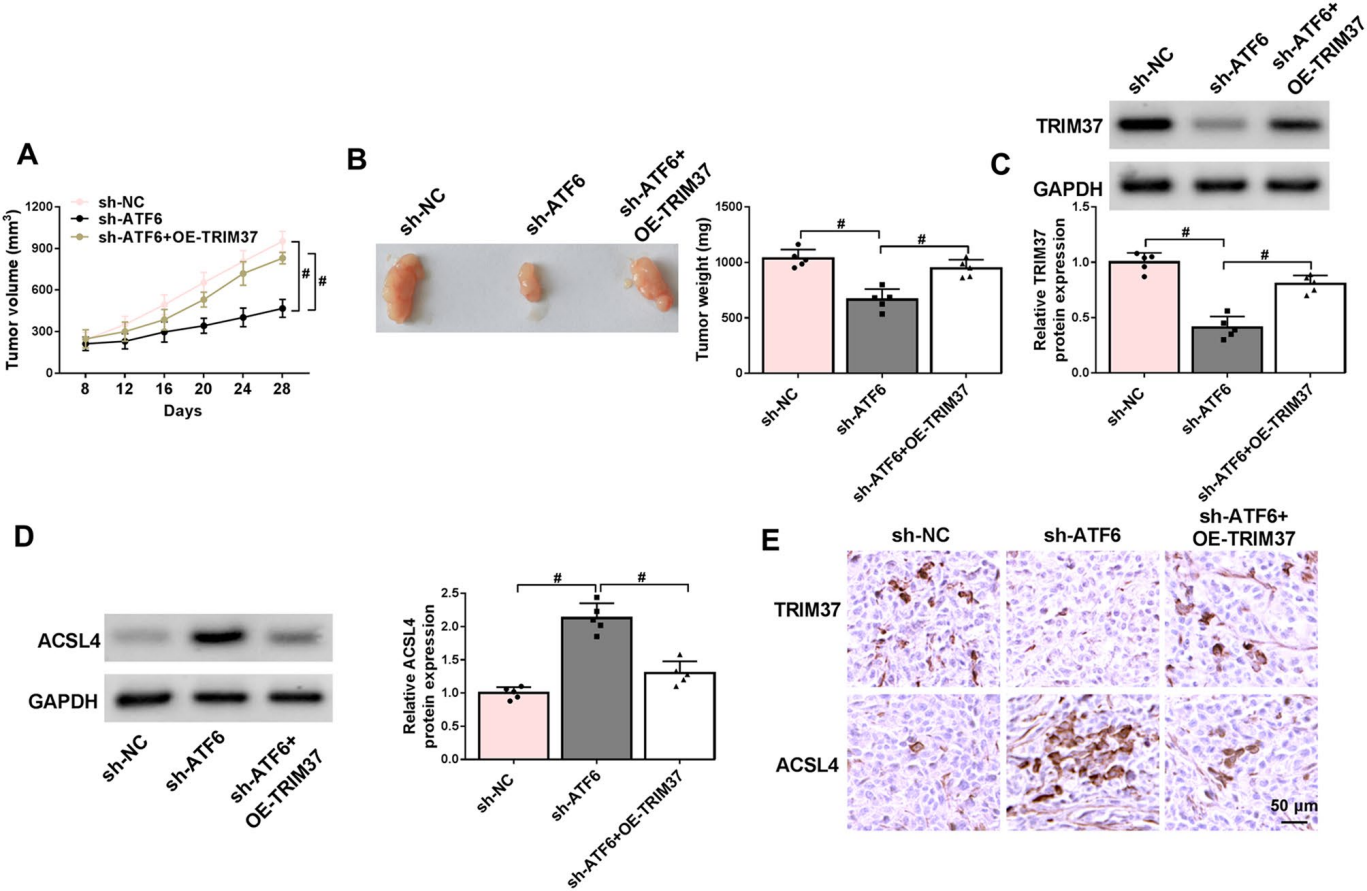
Overexpression of TRIM37 alleviated the impact of ATF6 knockdown on tumor growth. (**A**) Growth curves of CC xenografts in each group (sh-NC, sh-ATF6, and sh-ATF6 + OE-TRIM37). (**B**) Representative images and weights of CC tumors in each group. (**C**-**D**) Western blot analysis was applied to evaluate the expression levels of TRIM37 and ACSL4 in tumor tissues. (**E**) IHC was used to identify the expression of TRIM37 and ACSL4 proteins. # *P* < 0.05

Tumor volumes were measured every four days, revealing that tumor growth in the sh-ATF6 group was significantly slower compared to the sh-ATF6 + OE-TRIM37 group (Fig. 7A). The dissected tumor tissues were photographed and weighed. The growth-restraining consequence of ATF6 downregulation was annulled by TRIM37 overexpression (Fig. 7B). With the knockdown of ATF6, TRIM37 protein expression was reduced. Conversely, when ATF6 was knocked down, ACSL4 protein level was elevated. However, the expression level of ACSL4 declined with the upregulation of TRIM37 (Fig. 7C-D). Similarly, the results of IHC were consistent with those of Western blot (Fig. 7E). From the above, ATF6 promoted tumor growth by regulating TRIM37. The promoting effect exerted by ATF6 was achieved by facilitating the expression of TRIM37 to further suppress the expression of ACSL4.

## Discussion

Over the past few years, health education and HPV vaccination have become more widespread. As a result, there has been an obvious downward trend in the occurrence and fatality rates of CC globally [24, 29]. However, it is important to highlight that the average age of onset for new cases of CC is decreasing, indicating a trend toward young patients [35]. Since existing treatments are insufficient in comprehensively addressing these evolving complexities, discovering additional treatment targets has become a top priority [34]. These new targets could potentially unlock novel treatment strategies, although more research is needed to verify these findings and understand their broader applicability [45].

Abnormal regulation of ubiquitination in cancer development is thought to be an important mechanism in tumorigenesis and progression [27]. Ubiquitination modification has been demonstrated to play roles in the advancement of CC according to several studies [22, 26]. The research by Sun et al. has demonstrated that Parkin regulated IGF2BP3 through ubiquitination during the tumorigenesis of CC [38]. Research has also revealed that huperzine inhibited proliferation and promoted apoptosis in HeLa cells by affecting the ubiquitination degradation process of c-Myc protein [52]. As an important ubiquitinating enzyme, TRIM37 has been shown to be highly expressed in CC and to fuel cancer progression [37]. In line with this finding, our own research also reached a similar conclusion. The results found here provide novel insights by demonstrating that TRIM37 exhibits significantly elevated expression level in CC tissues and cells, confirming its role in cancer progression through rigorous experimentation. Moreover, in vitro cell experiments further demonstrated that the downregulation of TRIM37 could hamper the malignant behaviors of CC cells, indicating its crucial role in promoting cancer progression.

After thoroughly examining TRIM37’s function in the advancement of CC in the foregoing content, we further intend to explore how its upstream mechanisms precisely regulate the complex behaviors of TRIM37 in CC. Previous tumor studies have shown that ATF6 is a key factor determining the trends of tumor cell proliferation, apoptosis, and invasion and metastasis in solid tumors [2, 21, 42]. The research by Liu et al. discovered that ATF6 regulated the growth and migration of CC cells and repressed cell apoptosis and autophagy via the MAPK pathway [23]. However, transcriptional regulation of TRIM37 by ATF6 has not been reported. By using bioinformatics tools, we identified ATF6 as a potential transcription factor binding to TRIM37, with binding sites between their promoter regions. Experimental verification further confirmed that ATF6 could regulate TRIM37 expression in CC cells. Furthermore, the roles of both in CC cell behaviors and ferroptosis have also been explored. This finding indicates that the ATF6 - mediated regulation of TRIM37, which promotes CC progression, may have broader implications for other types of cancer expressing ATF6 and TRIM37.

Based on accumulating evidence, ACSL4-catalyzed arachidonic coenzyme A biosynthesis contributes to the execution of ferroptosis by triggering phospholipid peroxidation [9]. There is evidence demonstrating that ACSL4-mediated ferroptosis exerts an inhibitory effect on the growth and metastasis of CC [31]. As a key enzyme in the biosynthesis of polyunsaturated fatty acid-containing phospholipids, ACSL4 is known to be a central player in the execution of ferroptosis. Our experiments demonstrated that knocking down TRIM37 could promote the ferroptosis process of CC cells. Given the established role of ubiquitination in regulating protein stability and function, we hypothesized that TRIM37, with its ubiquitin-ligase activity, might interact with ACSL4 to modulate the ferroptosis process in CC cells. Strikingly, TRIM37 could modulate the protein level of ACSL4 without affecting its mRNA level. This finding suggested a post-transcriptional regulatory mechanism. TRIM37 likely acts through direct protein-protein interactions to target ACSL4 for ubiquitination. Moreover, TRIM37 indeed exerted its function by ubiquitinating ACSL4, and the ubiquitinated ACSL4 was subsequently degraded. In the ensuing experimental verification, the malignant cell proliferation triggered by TRIM37 downregulation was reversed by the downregulation of ACSL4. This result indicated that the reduced levels of ACSL4 could compensate for the loss of TRIM37-mediated oncogenic effects, suggesting a complex interplay between these two proteins in regulating cell growth. Moreover, the exacerbated ferroptosis of cells resulting from TRIM37 downregulation was also curbed by the knockdown of ACSL4. By modulating the protein level of ACSL4, TRIM37 can either promote or inhibit ferroptosis, depending on its expression status. Furthermore, in vivo investigations provided additional verification that ATF6 modulated the expression of TRIM37, consequently fueling the advancement of CC. Tumors with high levels of ATF6-induced TRIM37 expression exhibited enhanced growth and reduced ferroptosis, further validating the oncogenic role of this regulatory axis in the context of in vivo tumorigenesis.

Overall, we combined in vivo and in vitro experiments in this study to probe the molecular mechanisms underlying the development of CC. By interacting with its promoter, ATF6 boosts the expression of TRIM37. As a ubiquitinating enzyme, TRIM37 modifies the ferroptosis-related protein ACSL4 through ubiquitination, leading to its degradation. Thereby, this process inhibits cell ferroptosis and facilitates the malignant progression of CC. This research uncovers a novel molecular pathway involved in CC and highlights the therapeutic potential of targeting the ATF6-TRIM37-ACSL4 axis. Further research will focus on developing targeted therapies against the ATF6-TRIM37-ACSL4 axis, investigating its role in other cancer types, and conducting longitudinal studies to assess its clinical potential.

## Data Availability

No datasets were generated or analysed during the current study.

## Declarations

### Animal studies

Animal studies were performed in compliance with the ARRIVE guidelines and the Basel Declaration. All animals received humane care according to the National Institutes of Health (USA) guidelines.
